# Maladaptive and adaptive emotion regulation through music: a behavioral and neuroimaging study of males and females

**DOI:** 10.3389/fnhum.2015.00466

**Published:** 2015-08-26

**Authors:** Emily Carlson, Suvi Saarikallio, Petri Toiviainen, Brigitte Bogert, Marina Kliuchko, Elvira Brattico

**Affiliations:** ^1^Center for Interdisciplinary Music Research, Department of Music, University of JyväskyläJyväskylä, Finland; ^2^Cognitive Brain Research Unit, Institute of Behavioural Sciences, University of HelsinkiHelsinki, Finland; ^3^Helsinki Collegium of Advanced Studies, University of HelsinkiHelsinki, Finland; ^4^Advanced Magnetic Imaging (AMI) Center, Aalto UniversityEspoo, Finland

**Keywords:** music, emotion regulation, fMRI, prefrontal cortex, gender differences, mental health

## Abstract

Music therapists use guided affect regulation in the treatment of mood disorders. However, self-directed uses of music in affect regulation are not fully understood. Some uses of music may have negative effects on mental health, as can non-music regulation strategies, such as rumination. Psychological testing and functional magnetic resonance imaging (fMRI) were used explore music listening strategies in relation to mental health. Participants (*n* = 123) were assessed for depression, anxiety and Neuroticism, and uses of Music in Mood Regulation (MMR). Neural responses to music were measured in the medial prefrontal cortex (mPFC) in a subset of participants (*n* = 56). Discharge, using music to express negative emotions, related to increased anxiety and Neuroticism in all participants and particularly in males. Males high in Discharge showed decreased activity of mPFC during music listening compared with those using less Discharge. Females high in Diversion, using music to distract from negative emotions, showed more mPFC activity than females using less Diversion. These results suggest that the use of Discharge strategy can be associated with maladaptive patterns of emotional regulation, and may even have long-term negative effects on mental health. This finding has real-world applications in psychotherapy and particularly in clinical music therapy.

## Introduction

Although the ability of music to express and induce emotions seems so essential as to be obvious, it is only research from the last decades that allows us to state with scientific confidence that music can communicate and induce specific emotions to listeners (Balkwill and Thompson, 1999; Juslin, 2003; Juslin and Laukka, 2004; Juslin and Västfjäll, 2008; Fritz et al., 2009; Brattico and Pearce, 2013). This ability to induce emotions makes music listening a potentially powerful means of affect regulation, an important aspect of mental health, and could therefore have clinical applications. Indeed, music therapy is currently being used in the treatment of mental health disorders in clinical settings, with some evidence for its effectiveness, though there is a paucity of well-controlled studies to explain the mechanisms by which desired effects take place (Maratos et al., 2008). The need for improvements in current treatment of mental health disorders is not trivial. Current pharmacological treatment options, such as selective serotonin reuptake inhibitors (SSRIs) for depression, are far from perfect in their efficacy (Kirsch et al., 2008). The World Health Organization (WHO) has reported that up to 50% of those with psychiatric illness worldwide, including the perniciously common (and commonly comorbid) mood and anxiety disorders, do not receive adequate treatment (Demyttenaere et al., 2004), leaving a substantial gap that could be partially filled by the validation and development of music-based treatments, specifically for these more common disorders.

Music therapists are all too familiar with the need for clinical trials to support the use of such treatments, and most are equally familiar with the difficulties inherent in carrying out such trials, including gaining ethical permissions regarding vulnerable populations, finding financial means within stretched budgets, and simply having the time to consider research while meeting the demands of client care. Conducting clinical research with psychiatric clients can be especially difficult due to drop-out rates, distinctions between session attendance and active participation, and significant differences between treatment facilities where research may be conducted (Silverman, 2008). However, not all research need be done with clinical populations to be relevant to music therapy. In proposing a model of using research to develop therapeutic music interventions, Thaut (2002) emphasizes the importance of identifying shared and parallel brain functions between musical and non-musical psychological processes, allowing in turn for the development of musical experiences that have predictable therapeutic effects. Thaut further suggests that research regarding music therapy should follow four steps in order: (1) defining psychological, neurological and physiological responses to music; (2) defining non-musical processes which are functionally parallel to these musical responses; (3) defining the influence of music on non-musical responses and behaviors; and (4) defining the effects of music therapy on therapeutic outcomes (p. 116–119). The current study seeks to contribute to the first step of this research process, by defining parallels between non-musical and musical behaviors in affect regulation by examining parallels in outcomes between non-musical and musical strategies. We will also address the second step by examining neural responses to music in areas of the brain that have previously been associated with non-musical affect regulation processes, with a particular attention to maladaptive responses.

Given music’s ability to affect emotion, the most immediately relevant psychiatric disorders for which music may be explored as a therapeutic treatment are those overtly characterized by disruptions of normal affective functioning, such as clinical depression and anxiety (Juslin, 2003; Juslin and Västfjäll, 2008; Fritz et al., 2009). Following the terminology set by Juslin and Västfjäll (2008), this paper will use affect as an umbrella term to include both emotion and mood where possible for the sake of succinctness, still recognizing that mood and emotion are distinct yet interconnected both conceptually and functionally.

Affect regulation may be defined as a process by which an individual maintains or modifies his internal emotional or mood state, and includes behavioral and autonomic facets (Thayer et al., 1994). Deficits in an individual’s affect regulation ability, including the use of maladaptive strategies, have been linked with vulnerability to depression and anxiety (Fernandez-Berrocal et al., 2006; Gross et al., 2006; Gross and Thompson, 2007; Joormann and D’Avanzato, 2010; Joormann and Gotlib, 2010). Efficacious emotion regulation strategies, such as distraction and positive reappraisal, correlate negatively to undesirable outcomes such as depression (Oikawa, 2002; Gross and John, 2003; Garnefski et al., 2004), whereas inefficient emotion regulation strategies such as venting, suppression and rumination relate positively to depression and other mood disorders (Gross and John, 2003; Garnefski et al., 2004; Joormann and D’Avanzato, 2010). Habitual use of emotion regulation has furthermore been shown to affect neural responses to stimuli (Kanske et al., 2012). Previous research has found differences in how clinical psychiatric samples use music for affect regulation compared to non-clinical samples (Gebhardt and von Georgi, 2007; Gebhardt et al., 2014a,b).

Research has shown that there are differences between healthy and mentally ill populations in non-musical cognitive affect regulation strategies for dealing with negative stimuli. Cognitive reappraisal, a process of reassessing a stimulus as being less negative than originally perceived, has been associated with decreased risk of depression (Troy et al., 2010). Effective cognitive reappraisal is associated with increased activation of prefrontal and striatal areas in females, but with decreased amygdala response in males, suggesting important gender differences in affect regulation in the brain (McRae et al., 2008). Rumination, on the other hand, involves repetitive cognitive focus on the negative aspect of a situation, without attempts to alter the perception of the situation, and has been associated with the increased risk of depression and anxiety (Papadakis et al., 2006; Moulds et al., 2007; Arnone et al., 2009). Neuroimaging studies lend support to the existence of distinct neural affect regulation processes (Ochsner and Gross, 2007), and further more when habitual can affect neural responses to stimuli (Kanske et al., 2012). Differing regulation strategies, such as distraction or cognitive reappraisal, activate close but distinct regions of the prefrontal cortex (PFC), namely the lateral and medial prefrontal cortex (lPFC and mPFC), with the right lPFC and the mPFC, specifically the orbitofrontal cortex (OFC), increasing in activation when the regulation strategy requires a decrease of undesired emotion (Ochsner and Gross, 2005). Phillips et al. (2003) has posited two separate but related neural systems for emotional processing: a ventral system responsible for stimulus identification and automatic emotional responses, and a dorsal system responsible for emotional regulation. The PFC has also been shown to activate differentially in participants with depression when compared to healthy participants. Koenigs and Grafman (2009) found that the dorsal lPFC, associated with cognitive responses, was hypoactive in participants with depression, while the ventral mPFC was hyperactive, indicating neural irregularity in emotional regulation in depressed participants compared to healthy controls.

The process by which music can influence and regulate human affect is itself complex, and discovering the neural correlates of music-induced emotion and regulation is currently among the chief research interests in the field of music neuroscience (Levitin and Tirovolas, 2009; Brattico et al., 2013). Juslin and Västfjäll (2008) created a model that differentiates between six discreet but not mutually exclusive mechanisms by which music may change affect and which take place in distinct brain areas, with the amygdala, basal ganglia and inferior right frontal regions among those linked to the induction of basic emotions. Recently, Brattico et al. (2013) have proposed temporally organized model in which experience of discrete emotions, as observed in activation of structures including the amygdala, the anterior cingulate cortex (ACC) and PFC, follows early feature analysis at the brainstem and primary sensory cortical levels and cognitive processing (CP) of syntactic musical rules in non-primary sensory cortices and the ventral lPFC. Discrete emotional experiences, appearing in this model around 300 ms after the stimulus, precede aesthetic judgments and emotions and determinations of liking or disliking, which the authors propose may require listening to a piece of music in its entirety (p. 9).

Individuals make use of these responses to music to regulate their affective state in a variety of ways (von Georgi et al., 2006; Gebhardt and von Georgi, 2007; Saarikallio and Erkkilä, 2007; Gebhardt et al., 2014b). von Georgi et al. (2006) developed the Inventory for Measurement of Activation and Arousal Modulation (IAAM) scale using principal component analysis (PCA) of a battery of tests including a measure of psychological and psychopathological symptoms. Results identified relaxation (RX), CP, reduction of negative affect (RA), fun seeking (FS) and arousal management (AM) as key areas of music use in affect regulation (von Georgi et al., 2006). The IAAM has successfully been used to reveal differences between clinical psychiatric and non-clinical samples (Gebhardt and von Georgi, 2007; Gebhardt et al., 2014a,b). The Music in Mood Regulation scale (MMR) is a questionnaire that was derived originally from interview data with adolescents regarding how they used MMR, and developed through confirmatory factor analysis of a large sample of adolescents (Saarikallio, 2008). The MMR is an individual self-report measurement tool that defines seven categories of music mood regulation strategy, each defined by a typical mood prior to music use, type of music activity, social aspects, and changes in mood following music use (Saarikallio, 2008). The strategies defined by the MMR are: Entertainment, Revival, Strong Sensation, Diversion, Discharge, Mental Work and Solace. Diversion, Solace and Discharge are all related to using music to cope with negative mood states. Discharge is defined by a negative mood such as anger or sadness prior to music use leading the individual to listen to aggressive or sad music, the outcome of which is that the negative feelings has been expressed; it is most similar to the IAAM scale RA, which includes items such as “I listen to music when I really need to blow off steam” (von Georgi et al., 2006). Solace is similar to Discharge in that it is defined by a prior negative mood and listening to music that reflects the negative mood, but has an outcome of the listener feeling comfort, while Diversion is a strategy used by listeners wanting to be distracted from negative thoughts, with an outcome of successfully forgetting the current mood (Saarikallio, 2007, p. 96).

These differences suggests that some listening strategies may be more successful than others in achieving affect regulation, a possibility which in turn may have implications for music therapy. According to the Cochrane review on the subject, in the treatment of depression, a small number of clinical studies have shown that the addition of music therapy, including a variety of methods, can offer improved results compared to standard care alone (Maratos et al., 2008). Erkkilä et al. (2011) conducted a controlled trial and found that depression symptoms, anxiety symptoms and general functioning were significantly improved in clients who participated in 3 months of clinical music improvisation sessions compared to those who received only standard care. Similar results regarding music improvisation were found by Albornoz (2011). Other treatment approaches in music therapy in psychiatric settings include assisted RX, songwriting, lyric analysis and pairing music and movement (Silverman, 2007). Since a strong dose-response relationship has been shown in the effectiveness of music therapy treatment with psychiatric illness (Gold et al., 2009), and given some of the difficulties in providing regular and consistent access to active treatment described by Silverman (2008), appropriate use of specific listening strategies by clients outside of the therapy session could prove beneficial to therapeutic outcomes by increasing clients’ positive engagement with music. Such possible benefits might be parallel or complimentary to psychoeducational interventions, a meta-analysis of which by Donker et al. (2009) found to be effective in reducing anxiety and depression, and which recent study indicates may be as or even more effective in live music therapy contexts (Silverman, 2014, 2015). Furthermore, since many approaches to music therapy stress client-preferred music to music therapists in training as having the greatest benefit (Borczon, 2004), and client-preferred music has indeed been shown to be more effective in, for example, pain management (Mitchell and MacDonald, 2006), it would be imperative for a music therapist to know whether and how a client’s music listening outside of therapy might become maladaptive and even hinder treatment. The MMR, unlike the IAAM, has not yet been tested with clinical populations. However, since it distinguishes more finely between different ways listeners may use music to decrease negative affect, and because it was developed without regard to psychiatric symptoms, the MMR was considered to be more appropriate for this study, which is aimed at exploring the association of uses of music with maladaptive emotionality in the general population.

The topic of whether music-use might be unbeneficial or even counter-productive in therapy needs to be approached carefully, with an eye towards complexity derived from individual differences. In the past, concerns about adolescent suicide risks and violence lead to public speculation about the negative mental health effects of specific musical genres and the violence expressed in lyrics, but the studies resulted in mixed findings (Jones, 1997; Scheel and Westefeld, 1999; Lacourse et al., 2001), highlighting the danger of examining the relationship between music and mental health only in terms of the musical properties. Currently, a major shift in music research is taking place, with researchers acknowledging that the effects of music can not be understood only through the investigation of music as a stimulus but rather though investigating the ways that different individuals choose to engage with music (Garza Villarreal et al., 2011; Gold et al., 2013). Research now suggests that the selection of a particular affect regulation or coping strategy indeed plays an important role in defining the health-consequences of musical engagement (Miranda et al., 2012; Saarikallio, in press). Recent work has also shown that psychological features of the listener, such as stress reactivity (Thoma et al., 2012) tendencies for absorption and dissociation (Garrido and Schubert, 2011) and clinical depression (McFerran and Saarikallio, 2014), seem to moderate the connection between musical engagement and health-outcomes. For instance, some individuals listen voluntarily to sad music for pleasure, even when negative affect is sometimes experienced as a result, suggesting that personality traits affect both the likelihood that a person will choose to listen to sad music for pleasure, and whether the resulting affect is negative or positive (Garrido and Schubert, 2011, 2013). Research conducted with healthy populations typically suggests that listening to music that reflects one’s negative mood is a fundamentally healthy act, and serves as a means for solace (Saarikallio and Erkkilä, 2007), distraction and reappraisal (Van den Tol and Edwards, 2013), and gaining increased insight of the affective state (Skånland, 2013). Depressed adolescents, on the other hand, have been shown to be prone to couple their listening to sad and aggressive music with ruminative tendencies, social isolation and inability to improve their mood (McFerran and Saarikallio, 2014). Since the use of the MMR Discharge strategy in music listening does not tend towards mood repair, it might be considered analogous to rumination as maladaptive regulation behavior. In the MMR strategy Diversion, however, the individual uses music to distract herself from negative thoughts or emotions; the use of Diversion may therefore indirectly indicate less use of rumination, or even be considered its opposite.

The existing research thus allows us to define normal and maladaptive non-musical cognitive strategies for affect regulation, as well as normal and maladaptive non-musical neural processes of affect regulation. The goal of the current research is to further define maladaptive music listening strategies, as well as maladaptive neural responses to music, and explore connections between these two. We defined a maladaptive neural response to music as one that is associated with increased negative or decreased positive emotional experience when compared to normal listening responses, such as could be related to common affective psychiatric symptoms like prolonged negative mood. Because our study involved a normal, non-clinical sample of participants, we did not focus our attention on disruptions of neural responses related to emotion other than valence, such responses that could be considered mania or psychosis, as these were unlikely given our sample. Our goal, per Thaut (2002) model, was to define neural responses to music and parallels with non-musicalresponses, with special attention to behaviors and responses that might be maladaptive. We expected neural responses related to maladaptive emotion regulation could be seen in our non-clinical sample, as previous studies have found differences in neural activation in participants with sub-clinical levels of depression (Felder et al., 2012). The vmPFC and vlPFC, have been previously associated with non-musical affect regulation, including maladaptive regulation, leading us to hypothesize that neural correlates of maladaptive musical affect regulation would be visible in these areas. Specifically, our hypothesis was that relationships would exist between Discharge scores and increased depression, anxiety and trait neuroticism, the latter being considered as an established risk factor for mood disorders (Costa and McCrae, 1992; Hayes and Joseph, 2003). In turn, we hypothesized that there would be no such relationship with the MMR strategy Solace, despite the similarity of the strategies, and that Diversion strategy would also correlate negatively with mental illness and its risk factors and with maladaptive brain responses. Because previous literature has suggested that notable gender differences exist in affect regulation and neural responses between males and females, we chose to examine males and females separately in our analysis to prevent such possible gender differences from obscuring these exploratory results (Thayer et al., 2003; McRae et al., 2008; Mak et al., 2009; Joormann et al., 2012).

## Materials and Methods

### Behavioral Data Collection

#### Participants

A total of 123 participants (68 females), between the ages of 18 and 55 completed psychological testing. Participants’ mean age was 28.8 (SD = 8.89 years). Participants were recruited over a period of 18 months, from around the Helsinki area using fliers and email lists. The majority of these participants were non-musicians (*n* = 68), while others were identified as amateur musicians (*n* = 38) or professional musicians (*n* = 20). Sixty-two of these participants also provided socio-economic information, allow for the calculation of their socio-economic status as indicated by the H index score (Hollingshead, 1975), which ranged from 17 to 66 with a mean of 36.85 (SD = 18.25), with no significant differences between males and females *t*_(60)_ = 880, *p* = 0.382. The data collection, taking place in the arc of 15 months, was part of a larger project called Tunteet, including several experimental paradigms, psychological tests and even blood samples. Considering the complexity of the Tunteet protocol and the ability of participants to choose which parts of it to participate, not all the participants of the Tunteet project could be included in this study but only those for whom we obtained the relevant measurements. The full Tunteet protocol was approved by the local ethical committees of the Institute of Behavioural Sciences, University of Helsinki, and the Coordinating Ethical Board of the Uusimaa Hospital District.

#### Behavioral Testing

Participants completed self-report measures for assessing psychological functioning and musical engagement on paper and pencil. The Montgomery-Åsbert Depression Scale (MADRS) was used to test for levels of depression, the Neuroticism subscale of the Big Five Questionnaire (BFQ) to test for levels of Neuroticism, and the Anxiety facet of the Hospital Anxiety and Depression Scale (HADS-A) to measure levels of anxiety. Music-related affect regulation was measured using the MMR (Saarikallio, 2008), from which the subscales for strategies of Diversion, Discharge, and Solace were used in the current study to test our hypotheses.

The BFQ assesses the traits defined by the Five Factor Theory of Personality: Openness, Conscientiousness, Extraversion, Agreeableness and Neuroticism. Participants rank their level of agreement from on a five point Likert scale with statements related to each domain (Caprara et al., 1993). Only the subscale results of Neuroticism were used in this study as relevant to our hypothesis, being it associated to a risk of mental problems (Hayes and Joseph, 2003). For assessing depression we used the MADRS which is a diagnostic test, the scoring of which allows clinicians to rank depression levels based on the participants’ score between 0 and 60 points. Müller et al. (2000) correlated the MADRS to the Hamilton Depression Rating Scale in order to distinguish four levels of depression: none/recovered (1–8), mild (9–17), moderate (18–34), severe (>35) (Müller et al., 2000). Previous studies have also used the MADRS as a continuous measure (Raison et al., 2007). Although originally intended for clinical populations, previous studies have also used the MADRS to assess depressive symptoms in non-clinical populations, particularly at mild or subclinical levels (e.g., Unden et al., 2002; Van den Rest et al., 2008; Hidalgo et al., 2009; Sarkar et al., 2015). For anxiety assessment we used the HADS, a self-report measure designed to indicate the severity of depression and anxiety symptoms, and possible or probable cases of clinical disorders (Zigmond and Snaith, 1983) with demonstrated validity. In this study, the HADS was translated into Finnish from Swedish, resulting in some discrepancies in meaning, identified by native Finnish speakers. Because of this, only the Anxiety subscale (HADS-A) was used for this study. The HADS-A is scored from 0 to 21; presence of anxiety is classed as mild (8–10), moderate (11–14) or severe (>15). The HADS-A has also previously been used to measure anxiety symptoms in non-clinical (Carroll et al., 2000; Asbury et al., 2006; Van den Rest et al., 2008).

Psychological test scores were correlated to each other with parametric and non-parametric tests using SPSS version 22 (SPSS Inc., Chicago, IL, USA), running on Mac OS X 10.9.5.

### fMRI Data Collection

#### Participants

A subset of 63 participants also agreed to participate in an functional magnetic resonance imaging (fMRI) scanning session. Inclusion criteria were an absence of hearing or neurological problems, and psychopharmacological medication. Seven participants were excluded from the analysis due to technical issues, excessive movements during scanning, or neuro-radiological abnormalities. Recruitment was continuous until the desired number of participants for the experimental paradigm described below was reached. Of the remaining 56 participants (33 female) participants between the ages of 20 and 53 (mean age 28.5 years, SD = 8 years), eleven participants had played a musical instrument for at least 5 years, eight of whom continued to play music actively (at least 2 h/week). Twenty-nine of these participants also provided socio-economic information; their socio-economic status as indicated by the H index scores (Hollingshead, 1975) ranged from 17 to 66 with a mean of 33.62 (SD = 16.94), with no significant differences between males and females, *t*_(27)_ = −0.458, *p* = 0.651.

#### Paradigm

The music stimulus of 30 excerpts (10 each representing happiness, sadness, and fear) was derived from the Soundtracks dataset for music and emotion developed at the University of Jyväskylä by Eerola and Vuoskoski (2011), which is publically available for download online.^1^ Soundtrack music was considered appropriate for this study because it is composed with the intention of inducing emotional responses in listeners. For the current experiment, 10 representative excerpts were chosen for each of the happy, sad, and fearful categories, lasting 4 s each with 500 ms fade-in and fade-out. Short excerpts were considered appropriate for this study because previous studies have shown that emotion recognition occurs within 500 ms of hearing (Filipic et al., 2010), and have previously been used by Aubé et al. (2015). Both implicit and explicit processing of emotions conveyed through music was also considered, as affect regulation can be both a conscious and unconscious process (Thayer et al., 1994), resulting in six listening conditions in all, which are displayed in Table 1.

**Table 1:** **Listening conditions in fMRI study**.

Happy-implicit (HI)	Sad-implicit (SI)	Fear-implicit (FI)
Happy-explicit (HE)	Sad-explicit (SE)	Fear-explicit (FE)

Each participant underwent a single data collection session. In two blocks, each listened to the excerpts in a randomized order and completed a task of either identifying the emotion expressed by the music (explicit processing), or identifying how many instruments, they heard in the excerpt (implicit processing). Prompted by text on the screen, participants were given three answer choices in each task: “happiness, sadness or fear” in the explicit block, and “one, two or many” in the implicit block. The questions were presented orally via an intercom prior to each block and visually on the screen. The three answers were then presented on the screen and remained on the screen throughout the entire block. Each music excerpt was followed by a 5 s answer period, during which participants answered by pressing one of three push buttons on a response box.

#### fMRI Data Acquisition and Analysis

fMRI data was collected at the Advanced Magnetic Imaging (AMI) Center at Aalto University, using a 3 T MAGNETOM Skyra whole-body scanner (Siemens Healthcare, Erlangen, Germany). An interleaved gradient echo-planar imaging (EPI) sequence (TR = 2 s; echo time = 32 ms; flip angle = 75°) sensitive to blood oxygen level-dependent (BOLD) contrast was used to acquire 33 oblique slices allowing coverage of the whole brain (field of view = 192 × 192 mm; 64 × 64 matrix; slice thickness = 4 mm; spacing = 0 mm). Following the fMRI tasks, anatomical T1-weighted MR images (176 slices, field of view = 256 mm; 256 × 256 matrix; 1 mm × 1 mm × 1 mm; spacing = 0 mm) were collected.

Preprocessing and statistical analysis of fMRI data was performed using Statistical Parametric Mapping (SPM8; Wellcome Department of Imaging Neuroscience, London, UK) run using MATLAB (The MathWorks, Natick, MA, Inc.). Images for each participant were realigned to adjust for movement between slices, normalized spatially onto the Montreal Neurological Institute (MNI) template (6 parameters rigid body model, gray matter segmentation), and spatially smoothed using a Gaussian filter with an FWHM of 6 mm. Brain volumes were then screened to determine whether they met the criteria for high quality and scan stability as determined by small motion correction (<2 mm translation and <2° rotation). Data was filtered temporally at 128 Hz to minimize artifacts caused by the scanner. fMRI responses were modeled using a canonical, hemodynamic response function (HRF), and the six movement parameters were used as regressers of no interest.

Using SPM8, beta estimates for each participant’s average BOLD response at each voxel were computed, and *t*-contrasts were used to compare whether a condition elicited a different brain activation compared to another. The BOLD response for each condition compared to baseline was computed for each participant.

**Figure 1:**
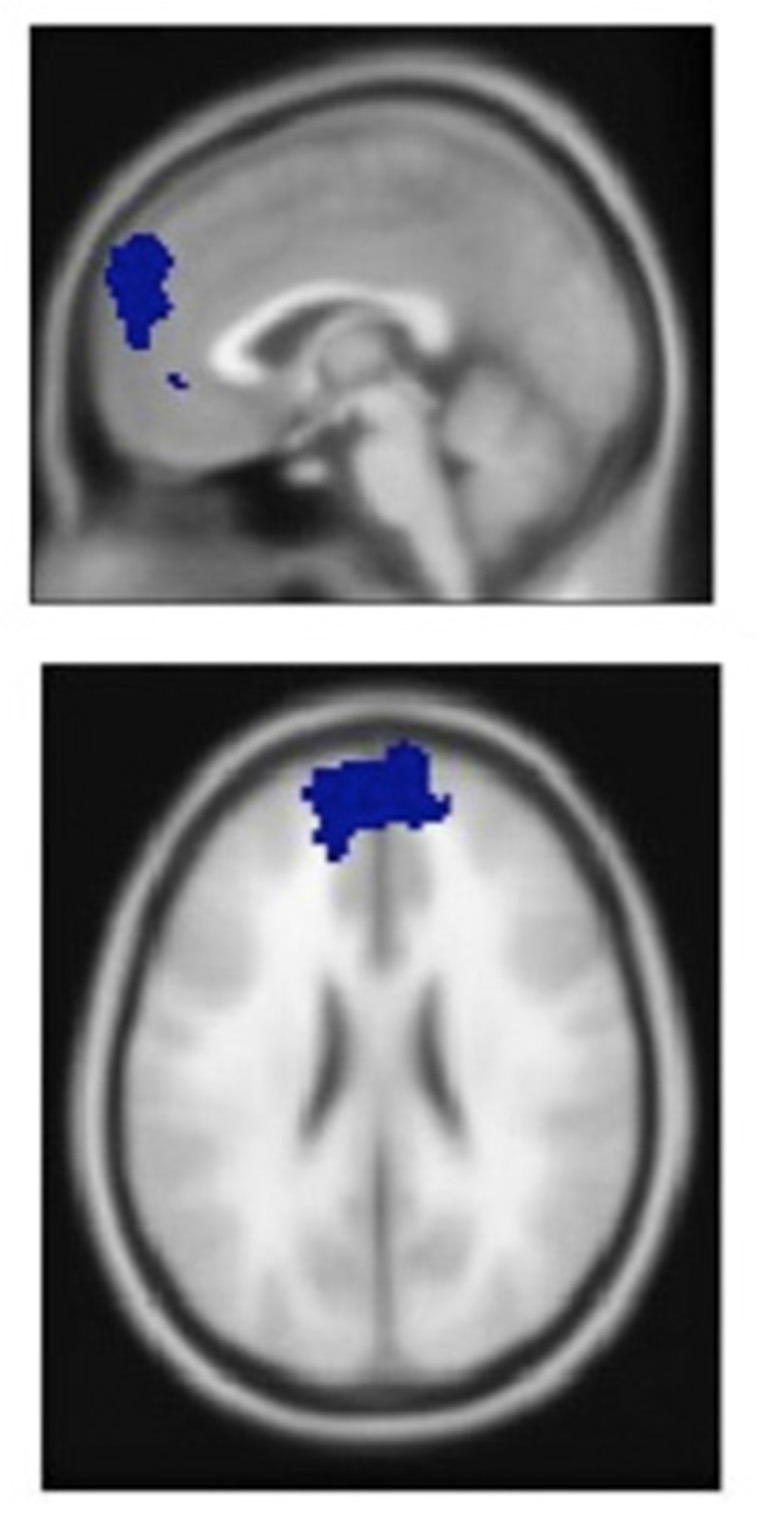
**Medial prefrontal cortex (mPFC) area identified by general linear model (GLM) analysis of neural activation during music listening**.

To test the relation between emotion regulation in the brain and music regulation strategies, we focused our analysis on the PFC, in which emotional processes are controlled and regulated (Ochsner and Gross, 2005; Koenigs and Grafman, 2009). Regions of interest (ROIs) were calculated using the MarsBaR Toolbox (Brett et al., 2002). General linear model (GLM) analysis, done by Bogert et al. (submitted) showed significant activity in a large ROI centered on the mPFC (Figure 1). We used activity in this ROI for further analysis in combination with the behavioral data.

Data were analyzed using the SPSS statistical package. Behavioral data was explored using correlational analysis. In order to reduce the number of tests performed, we used ANOVA rather than correlation analysis to explore the relationships between behavioral and fMRI data, first in all participants and then separately in males and females. Mixed design ANOVAs were used, with three levels of music emotion (happy, sad or fear) and two levels of processing type (implicit or explicit) as within-participant factors, and high- and low-scoring groups according to MMR scores as between-participant factors. Two-tailed *t*-tests were used for more detailed assessment of between-participant effects in males and females separately.

## Results

### Behavioral Data

MADRS, HADS-A and Neuroticism (as measured by the BFQ) results revealed a statistically normal, generally psychologically healthy sample. Twenty-three participants (19%) scored between 9 and 17 on the MADRS, meeting scale criterion for mild depression. Three participants scored above 20 on the MADRS, and could thus be considered moderately depressed. Eighteen participants (16%) scored between 8 and 10 on the HADS-A, and could thus be considered to have mild anxiety. Only one participant scored above 12, and could thus be considered to have moderate anxiety. Internal reliability of all tests was good, with Cronbach’s *α* ranging between 0.70 and 0.85. The results and reliability scores of all tests are displayed in Table 2.

**Table 2:** **Test results for all participants (*n* = 123), males (*n* = 55) and females (*n* = 68)**.

	Cronbach’s *α*	All participants		Males		Females	
		*M*	SD	*M*	SD	*M*	SD
MADRS	0.77	5.37	4.55	5.27	3.70	5.45	5.17
HADS-A	0.72	4.80	2.71	5.00	3.10	4.65	2.35
Neuroticism	0.85	17.36	7.59	15.43*	7.20	19.13*	7.84
Discharge	0.70	2.52	1.06	2.55	1.07	2.49	1.06
Diversion	0.78	3.07	0.89	2.88*	0.78	3.22*	0.96
Solace	0.82	3.36	1.02	3.33	1.06	3.37	0.98

*Significant difference between males and females, *p* < 0.01.

Results of all tests were correlated in all 123 participants. As expected, MADRS scores were significantly positively correlated with HADS-A scores, *r* = 0.53, *p* < 0.001, and with Neuroticism scores, *r* = 0.54, *p* < 0.001. Neuroticism correlated strongly with HADS-A scores, *r* = 0.62, *p* < 0.001. MMR Discharge scores were weakly but significantly correlated with HADS-A scores, *r* = 0.24 *p* = 0.007, and similarly correlated with Neuroticism scores, *r* = 0.20, *p* = 0.02. Neither MMR Diversion nor Solace correlated with MADRS, HADS-A, or Neuroticism scores.

Correlations were also determined for male and female participants separately to explore possible idiosyncrasies in uses of music and maladaptive emotionality depending on gender. Independent sample *t*-tests showed that there was a significant difference between females and males in Neuroticism scores, with females (*M* = 19.13, SD = 7.20) having a higher mean score than males (*M* = 15.43, SD = 7.84); *t*_(119)_ = −2.69, *p* = 0.008. There was also a significant difference in MMR Diversion scores between females (*M* = 3.22, SD = 0.962), and males (*M* = 2.88, SD = 0.780); *t*_(119)_ = −2.07, *p* = 0.04, with higher scores for females. There were no other significant differences between genders; in Discharge use, males (*M* = 2.56, SD = 1.06) were only slightly higher than females (*M* = 2.48, SD = 1.07).

**Figure 2:**
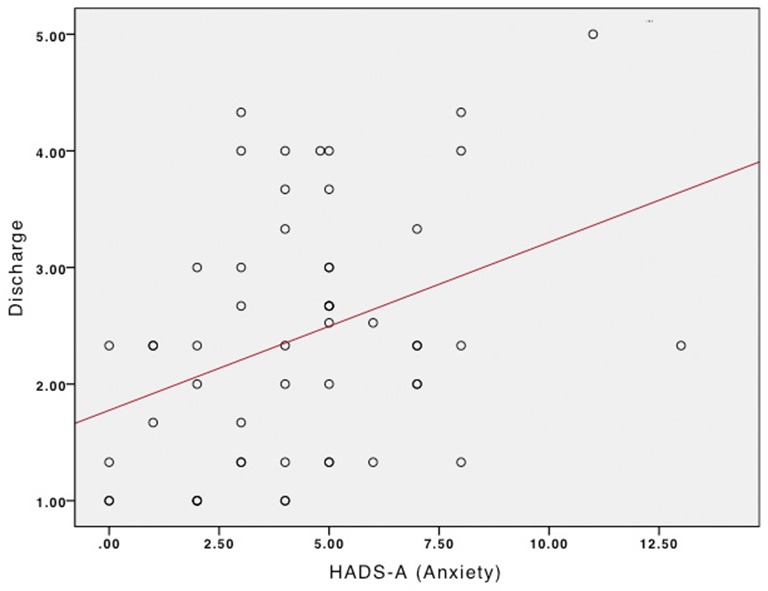
**Relationship between Anxiety and Discharge in males**.

**Figure 3:**
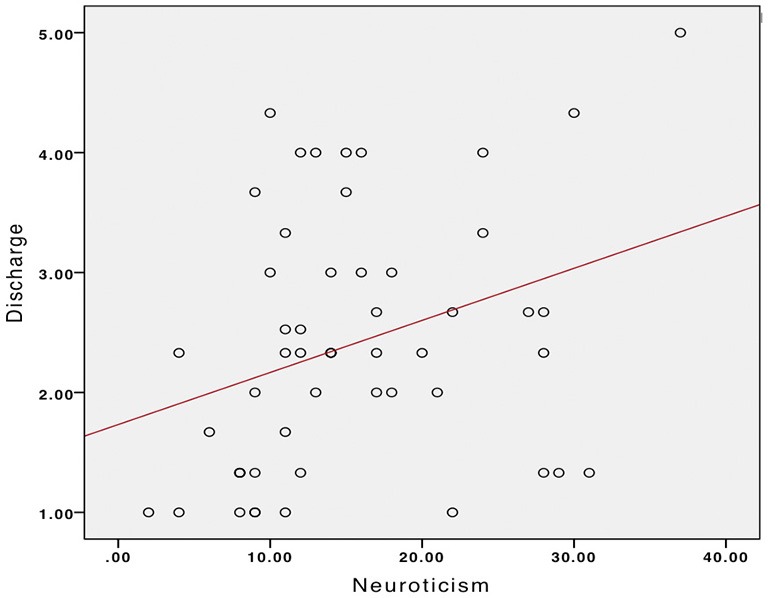
**Relationship between Neuroticism and Discharge in males**.

Based on the gender differences in emotionality and in uses of music obtained in the *t*-tests, we performed separate correlation tests to explore associations between uses of music and emotional profiles in males and females. There were no significant correlations for female participants between MMR scores and other psychological scores. For male participants, positive correlations were found between HADS-A scores and MMR Discharge, *r* = 0.36, *p* = 0.007 (Figure 2), and between Neuroticism and MMR Discharge, *r* = 0.32, *p* = 0.02 (Figure 3).

### Relationships Between fMRI and Behavioral Data

Since only Discharge correlated with anxiety and neuroticism scores in the previous analysis, and since only Diversion was found to be significantly different by gender, only these two strategies were included in further analysis. *T*-tests showed no significant differences in MADRS, HADS-A, Neuroticism or MMT scores between this subset and the rest of the group (*p* ranged from 0.38 to 0.93), suggesting that this group could fairly be considered representative of the whole. In this group, however, there was no significant difference between Neuroticism scores, *t*_(1,54)_ = −0.62, *p* = 0.53.

Participants were divided into high and low-scoring groups based on the median scores for Discharge and Diversion. Mixed design ANOVAs were performed on the data, using the three music emotions and the two processing types as within-participant factors; one test with high and low Discharge scorers as between participant groups, one test with high and low and Diversion scorers as between-participant factors, to compare differences in activation in the mPFC. Participants were divided into high scoring and low scoring groups to determine whether high or low scores had a significant effect on mPFC activation. No significant relationships were found between MMR scores and mPFC activation when including all participants in the ANOVA analysis. However, since previous literature has found significant differences between genders in neural activity during emotion regulation (Thayer et al., 2003; McRae et al., 2008; Mak et al., 2009; Joormann et al., 2012), and since our own analysis showed differences between males and females in Diversion use, and correlations with anxiety and Neuroticism with Discharge for males but not females, we conducted further analysis for males and females separately.

ANOVA results showed that, for mPFC activation, there was a significant main effect of Diversion score (low or high) in both females (*F*_(1,31)_ = 5.66, *p* = 0.03) and males (*F*_(1,21)_ = 5.34, *p* = 0.03). There was also a significant main effect of Discharge score on mPFC activation for males (*F*_(1,21)_ = 8.65, *p* = 0.04) but not for females. Figure 3 shows activation levels of the mPFC during the six different listening conditions for females and males separately, divided by high and low scores in Discharge and Diversion.

**Figure 4:**
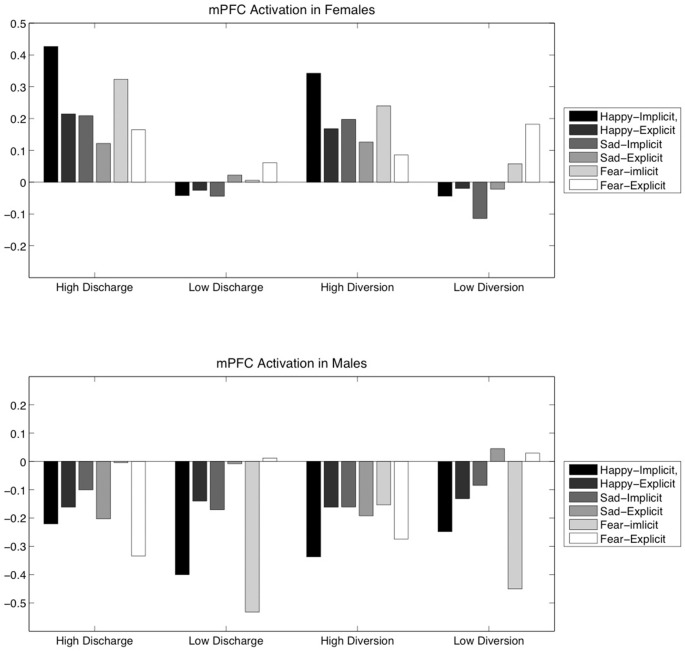
**High- and low-score groupings of female and male participants based on either Discharge or Diversion score show differences in mPFC activation, which is shown above by listening condition**.

Further detail was obtained by performing a series of two-tailed *t*-tests (see Figure 4), comparing the mPFC activity in low and high scorers in Discharge and Diversion within each of the six listening conditions, with males and females again analyzed separately. To reduce the number of tests performed, only Diversion was tested in females, since Discharge was not significant in the ANOVA. Two-tailed *t*-tests showed a significant difference in mPFC activity between females scoring high or low in Diversion use during the implicit listening condition for all three types of music: happy, (*t*_(31)_ = −3.11, *p* = 0.004) sad, (*t*_(31)_ = −2.23, *p* = 0.03), and fearful (*t*_(31)_ = −2.46, *p* = 0.02). These results indicate that females who had higher scores in Diversion experienced higher mPFC activity to emotional music.

For Discharge use in males, in turn, we did not obtain any significant differences in two-tailed *t*-tests. Instead, for Diversion use in males, we found a significant difference in the mPFC activation to fearful music in the explicit listening condition, *t*_(21)_ = 2.56, *p* = 0.02. However, it can be noted that, under all conditions, males who more frequently used Discharge or Diversion had a general tendency towards decreases in mPFC activation during music listening.

## Discussion

The aim of this study was to explore potentially maladaptive behaviors and brain responses in the domain of music listening by defining brain responses to music related to mood regulation, and to exploring relationships between music mood regulation strategies and mental health outcomes. Our results suggest that the use of music as affect regulation is subject to individual differences, including differences such as anxiety, depression and Neuroticism levels that are also related to mental health. Furthermore, the results of this study highlight significant differences between gender both in behavior and neural responses to music in relation to affect regulation. These findings may be useful in guiding music therapy practice, particularly in terms of developing supplemental, psychoeducational methods, as well as informing future research into this area.

### Behavioral Measures

The results of this study indicate a weak positive correlation between participant scores in the music mood regulation strategy Discharge, Neuroticism and anxiety measures in all participants. This effect is comparable in strength to significant results found by Gebhardt et al. (2014a, p. 488), which showed an increased tendency to use music for RX in psychiatric patients with lower functioning. Although the use of music for RX and the Discharge strategy are not comparable, the results are similar in their suggestion that increased psychological distress may lead individuals to seek relief through music listening more often.

There were significant differences between male and female scores in Neuroticism, in which females scored significantly higher than males as predicted by previous literature (Costa et al., 2001). There were also significant differences between males and females in the use of Diversion as a music mood regulation strategy, with females using music for Diversion more than males. According to Saarikallio (2007), adolescents described using Diversion as a regulatory tacit to prevent their minds from straying to negative or anxious thoughts (p. 99). One explanation for this may be that females are more likely to employ avoidance coping strategies than males (Matud, 2004).

No significant correlations were found between Discharge and depression scores in all participants or when divided by gender. This may be because Discharge, defined as it is by the *expression* of negative emotion, might be considered an externalizing behavior, while depression is often categorized as an internalizing pathology (Miranda et al., 2012). This may also, however, be because the participants in this study were generally not depressed, or had only subclinical (mild) levels of depressive symptoms, and a clinically diagnosed sample may prove more revealing in this regard. That Discharge was significantly correlated with both anxiety and Neuroticism suggests a relationship with negative mental health outcomes. That one of its characteristics is that sad or angry music is heard, along with its correlation with anxiety and Neuroticism, may suggest a similarity between Discharge and rumination, a maladaptive strategy.

The possibility that Discharge is a maladaptive strategy is further strengthened by the fact that Solace and Diversion were *not* correlated with anxiety and Neuroticism, though they too, are associated with negative affect prior to musical engagement. It is possible that Solace and Diversion are better mood regulation strategies than Discharge, and may even act as moderating or protective factors in the relationship between music listening and mental health risk. Gebhardt and von Georgi (2007) finding that clinical patients with affective disorders specifically use music for negative affect reduction less than others, may also support the interpretation that positive and effective music listening can have positive effects on mental health and its lack can reflect pathology. However, much more research would be needed to determine causation (Miranda et al., 2012).

These correlations persisted for males but not females when participants were examined by gender. McRae et al. (2008) suggested that affect regulation might be a more automatic process for males than for females. Although Discharge is not a suitable musical analog for CRA, it is worth considering that a person for whom affect repair is less conscious may be more likely to chose music to express rather than actively repairs negative mood; that is, chose Discharge over Solace or Diversion. Similarly, if affect regulation is more conscious for females, a female may be more likely to actively choose to divert attention away from negative affect. Knight et al. (2002) found that males experience greater emotional arousal and greater difficulty regulating that arousal in response to aggressive-relevant stimuli compared to females. Thus, males who chose to listen to aggressive music while in a negative affective state may indeed be prone to prolonging the negative state more than females. This suggests that, as a possibly maladaptive strategy, Discharge may be prevalent and more harmful in males.

### fMRI Results

Because a relationship between music use and risk for mental disorders was suggested by the results above, we further investigated Discharge and Diversion in relation to brain activity. Our aim was to explore neurological correlates of the use of these strategies. We focused our attention on the mPFC, which previous literature has shown to be active in the processing of emotional stimuli (Phan et al., 2005; Vuilleumier, 2005), and in the active in the suppression of negative mood (Ochsner and Gross, 2005; Phan et al., 2005).

Mixed design ANOVAs showed significant main effect of Diversion on mPFC activation in both females and males, and of Discharge on mPFC activation in males only. *T*-tests revealed that females with higher Diversion scores had higher levels of activation in the mPFC during music listening. Since PFC hypoactivity has been associated with depression (Koenigs and Grafman, 2009), including in subclinical and remitted cases (Kanske et al., 2012; Sarkar et al., 2015), mPFC increased activation in female participants who scored higher on Diversion lends support to the idea that Diversion may be considered an effective, healthy listening strategy.

The only significant *t*-test for male participants showed that males who scored highly in Diversion use had significantly lower activation of the mPFC while explicitly attending to fearful music. However, the significant result of the ANOVA for males based on Discharge despite no significant *t*-tests, and the trend towards lower mPFC activation in high scorers in Discharge, suggests a broad tendency of Discharge to be associated with decreased mPFC activity in males. The same tendency, however, was observed in male high scorers in Diversion. This may present a problem for the hypothesis that Diversion is an effective form of music mood regulation. It may also point to importance gender differences in emotion processing and affect regulation. Males have previously been shown to have different neural responses to fearful stimuli compared to females (Schienle et al., 2005). Males have also been shown to have less PFC activation compared to females during affect regulation (McRae et al., 2008). More research is needed to clarify the role of gender in the current results.

That *t*-tests were generally not significant for male participants, although ANOVA results were significant, may suggest that the listening task was less determinant of males’ mPFC activation than other factors, including Discharge use, which would be in line with previous research showing neural responses differences based on habitual emotion regulation strategy (Kanske et al., 2012). However, because here we only used questionnaires rather than an experimental manipulation, it cannot be concluded whether these neural underpinnings are caused by repeated Discharge, anxiety and Neuroticism, or some other factor common to all three.

### Music Therapy and Future Research

Espousing the philosophy that basic music psychology research can be used to support, strengthen and inform clinical music therapy practice (Thaut, 2002), the results of the current study has aimed to contribute information about neural responses to music, non-clinical behavioral and cognitive engagement with music, and their respective relationships with mental health outcomes. The current results may be particularly applicable to psychoeducational treatment methods. However, further research is needed to determine the applicability of these results to other forms of music therapy.

Further research would of course be required to determine a causal link between Discharge use and mental health risk. Other types of research, including longitudinal study and qualitative investigations, could provide deeper and more nuanced understanding of the role of Discharge in psychological processes related to mental illness and mental health. Study involving clinical samples, and with careful controls for gender, could add much to the current results in terms of understanding the relative efficacy of MMR listening strategies. Finally, specific clinical music therapy studies are imperative to determining the efficacy of music therapy in applying these and further findings.

### Limitations

Several limitations need to be noted regarding the current study. First, there were no explicit measures of non-musical mood regulation behavior in the participants; connections between MMR results and non-musical types of affect regulation have been therefore be based on theory and previous findings, where empirical findings would be stronger and should be a topic of further study. As this study only examined neural responses in the mPFC, it likely presents an incomplete picture of the relationship between music mood regulation strategies and neural responses to music. Further study could also include clinical samples, which would shed more light on therapeutic applications of the use of music for mood regulation. As this sample was recruited only from the Helsinki area, it is possible that cultural homogeneity had some influence on results, although there are similarities in the CP of music across cultures (Krumhansl et al., 2000). The relatively young sample also limits the generalizability of the results for older populations. However, as suggested by Gebhardt and von Georgi (2007), the use of music to increase positive affect may be a behavior associated with younger populations in general, thus allowing the current findings to be, if not completely generalizable, acceptably relevant. The presence of professional musicians in the sample, as well as the broad recruitment measures, should also be taken into consideration in terms of the generalization of the results. Because, as McRae et al. (2008) showed, the PFC is differently activated in males compared to females during affect regulation, it cannot be said for certain whether these results merely reflect gender differences rather than Discharge-use, anxiety or Neuroticism. Another limitation is that, to avoid Type II errors, we chose to limit the number of statistical tests performed as much possible rather than to correct for multiple comparisons, which may of course have resulted in Type I errors. However, as there is theoretical support for our findings, we believe that they are unlikely to be largely spurious.

## Conclusion

This study has shown the possibility that an individual’s use of music, particularly in response to negative affect, may relate to his or her mental health, as evidenced by correlations between listening tendencies as mental health outcomes. These results suggest Discharge may be an ineffective or harmful listening strategy in response to negative affect, while Solace and Diversion may provide more effective mood regulation. This result may encourage music therapists to explore healthy music-based affect regulation with their clients, and to help clients identify ineffective or harmful listening strategies, and to develop and inform active interventions based on these identified cognitive strategies of music listening.

These results suggest that the mPFC is one key area in the processing music-emotions, and is activated more strongly in females who tend to use Diversion, but was decreased in males who scored highly on Discharge during the listening task overall. These differences point to possible neuro-mechanisms explaining the relative efficacy of Diversion and opposed to Discharge as an affect regulation strategy, implicate gender differences that may be reflective of different neural or psychological functioning of males and females, and require careful exploration future research.

## Conflict of Interest Statement

The authors declare that the research was conducted in the absence of any commercial or financial relationships that could be construed as a potential conflict of interest.

## Footnotes

^1^https://www.jyu.fi/hum/laitokset/musiikki/en/research/coe/materials/emotion/soundtracks/
